# Genetic Characterization of *Cryptosporidium cuniculus* from Rabbits in Egypt

**DOI:** 10.3390/pathogens10060775

**Published:** 2021-06-20

**Authors:** Doaa Naguib, Dawn M. Roellig, Nagah Arafat, Lihua Xiao

**Affiliations:** 1 Department of Hygiene and Zoonoses, Faculty of Veterinary Medicine, Mansoura University, Mansoura 35516, Egypt; doaanaguib246@yahoo.com; 2 Division of Foodborne, Waterborne, and Environmental Diseases, National Center for Emerging and Zoonotic Infectious Diseases, Centers for Disease Control and Prevention, Atlanta, GA 30329, USA; iyd4@cdc.gov; 3 Department of Poultry Diseases, Faculty of Veterinary Medicine, Mansoura University, Mansoura 35516, Egypt; nagaharafat@yahoo.com; 4 Center for Emerging and Zoonotic Diseases, College of Veterinary Medicine, South China Agricultural University, Guangzhou 510642, China

**Keywords:** *Cryptosporidium cuniculus*, rabbits, Egypt, *gp60* gene, PCR-RFLP, zoonoses

## Abstract

Rabbits are increasingly farmed in Egypt for meat. They are, however, known reservoirs of infectious pathogens. Currently, no information is available on the genetic characteristics of *Cryptosporidium* spp. in rabbits in Egypt. To understand the prevalence and genetic identity of *Cryptosporidium* spp. in these animals, 235 fecal samples were collected from rabbits of different ages on nine farms in El-Dakahlia, El-Gharbia, and Damietta Provinces, Egypt during the period from July 2015 to April 2016. PCR-RFLP analysis of the small subunit rRNA gene was used to detect and genotype *Cryptosporidium* spp. The overall detection rate was 11.9% (28/235). All 28 samples were identified as *Cryptosporidium cuniculus*. The 16 samples successfully subtyped by the sequence analysis of the partial 60 kDa glycoprotein gene belonged to two subtypes, VbA19 (*n* = 1) and VbA33 (*n* = 15). As *C. cuniculus* is increasingly recognized as a cause of human cryptosporidiosis, *Cryptosporidium* spp. in rabbits from Egypt have zoonotic potential.

## 1. Introduction

Cryptosporidiosis is a common cause of diarrhea in humans and animals [1,2]. It is one of the most important diseases in both developing countries and industrialized nations due to its importance in diarrhea-associated death in young children and foodborne and waterborne outbreaks of illness [1,3,4,5,6]. The etiologic agents of cryptosporidiosis, *Cryptosporidium* spp., have over 40 established species and many genotypes of unknown species status [7]. Among them, approximately 20 species and genotypes have been found in humans [8]. Most human cryptosporidiosis cases are caused by *C. parvum* and *C. hominis*. Other human-pathogenic *Cryptosporidium* spp. include *C. meleagridis*, *C. ubiquitum*, *C. cuniculus*, *C. felis*, *C. canis*, *C. viatorum*, and *C. muris* [7]. 

Rabbits are a supply of high-quality protein to humans. Reports of the Food and Agriculture Organization of the United Nations (FAO) showed that Egypt was the fourth largest producer of rabbit meat in the world, with approximately 7.6 million rabbits [9,10]. Results of recent studies indicate that rabbits can serve as reservoirs of many zoonotic pathogens [11,12,13]. They are commonly infected with several *Cryptosporidium* species, especially *C. cuniculus* [13,14,15,16,17,18,19,20]. In recent years, there have been increasing reports of *C. cuniculus* in humans [21,22,23,24]. In the United Kingdom and New Zealand, *C. cuniculus* is the third most common *Cryptosporidium* species in patients with diarrhea [24,25]. 

In recent years, molecular epidemiological studies have been conducted to understand the transmission of *Cryptosporidium* spp. in humans, livestock, and companion animals in Egypt [26,27,28,29,30,31,32,33,34]. Although rabbits are commonly farmed in Egypt, to the authors’ knowledge there have been no thorough studies on the distribution and genetic identity of *Cryptosporidium* spp. in rabbits in the country. Therefore, this study was conducted to examine the occurrence, genetic characteristics, and zoonotic potential of *Cryptosporidium* spp. in rabbits from three provinces (El-Dakahlia, El-Gharbia, and Damietta) in Egypt.

## 2. Results

### 2.1. Cryptosporidium Infection on Rabbit Farms 

*Cryptosporidium* spp. were detected by PCR analysis of the SSU rRNA gene in 28 (11.9%) of the 235 fecal samples analyzed in the study. Eight of the nine farms examined were positive for *Cryptosporidium* spp. Among the eight positive farms, the infection rates ranged from 4% to 24% (Table 1). The farms El-Dakahlia 3 and El-Gharbia 2 had high infection rates of 21% and 24%, respectively, while no infection was detected on farm El-Gharbia 1. By age, *Cryptosporidium* spp. were detected in rabbits of all ages, with a higher infection rate found in rabbits of <3 months (20%; Fisher’s exact test = 11.237, *p* = 0.003 in the overall comparison) (Table 2). By animal breed, *Cryptosporidium* spp. were identified in all breeds, with slightly higher detection rates in Hi-Plus rabbits (15%) than in New Zealand (11%) and Rex (7%), although these rates were not statistically different (Fisher’s exact test = 2.283, *p* = 0.333 in the overall comparison). Farms in El-Dakahlia province recorded higher *Cryptosporidium* occurrence (17%) than El-Gharbia (11%) and Damietta (7%) provinces (Fisher’s exact test = 3.685, *p* = 0.157 in the overall comparison).

### 2.2. Cryptosporidium Genotypes and Subtypes

**Figure 1:**
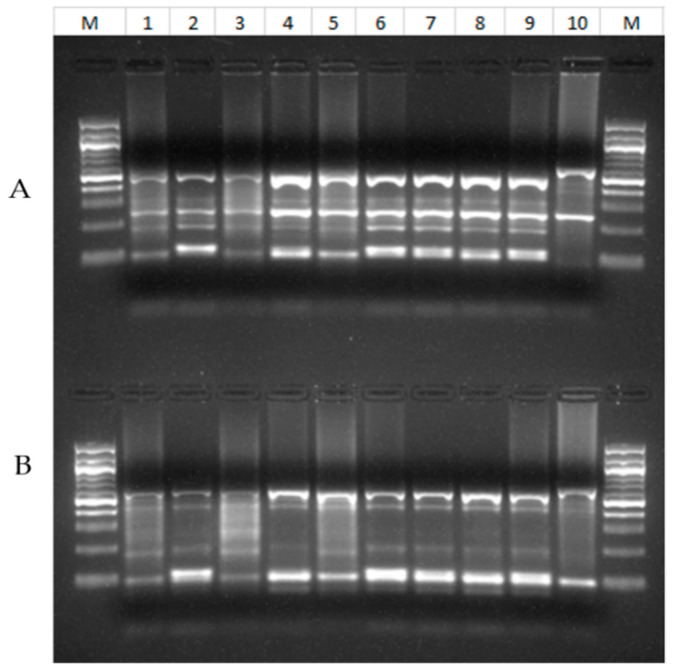
RFLP analysis of PCR products of *SSU rRNA* gene from *Cryptosporidium cuniculus* from rabbits using *Ssp*I (**A**) and *Vsp*I (**B**) restriction enzymes. Lane M: 100 bp molecular markers; Lane 1–9: *C. cuniculus*; Lane 10: positive control (*C. baileyi*).

**Figure 2:**
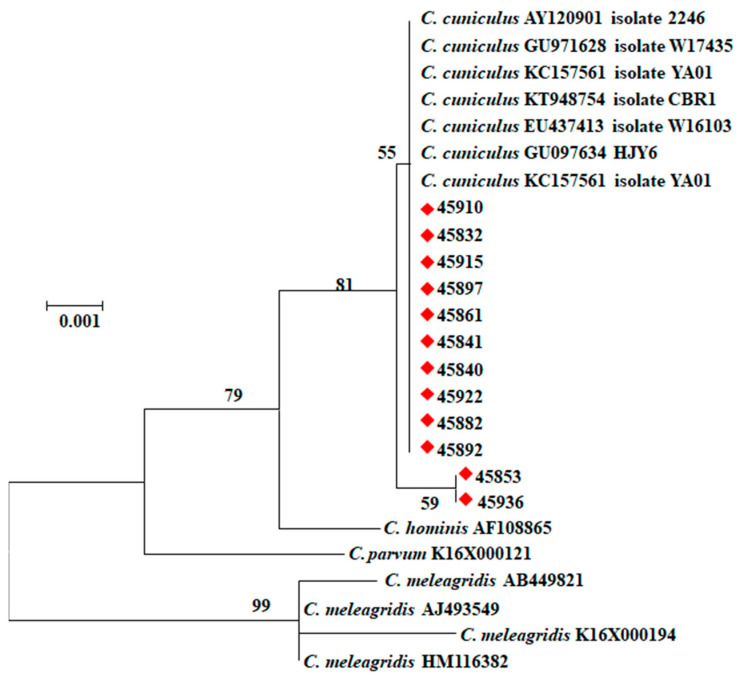
Phylogenetic relationships among *Cryptosporidium* spp. based on the nucleotide sequences of the *SSU* rRNA gene through a maximum likelihood analysis based on substitution rates calculated with the general time reversible model. Numbers at the internodes represent bootstrap values (>50%) from 1000 replicates. The *Cryptosporidium cuniculus* samples identified in this study are labeled with red rhombus.

**Figure 3:**
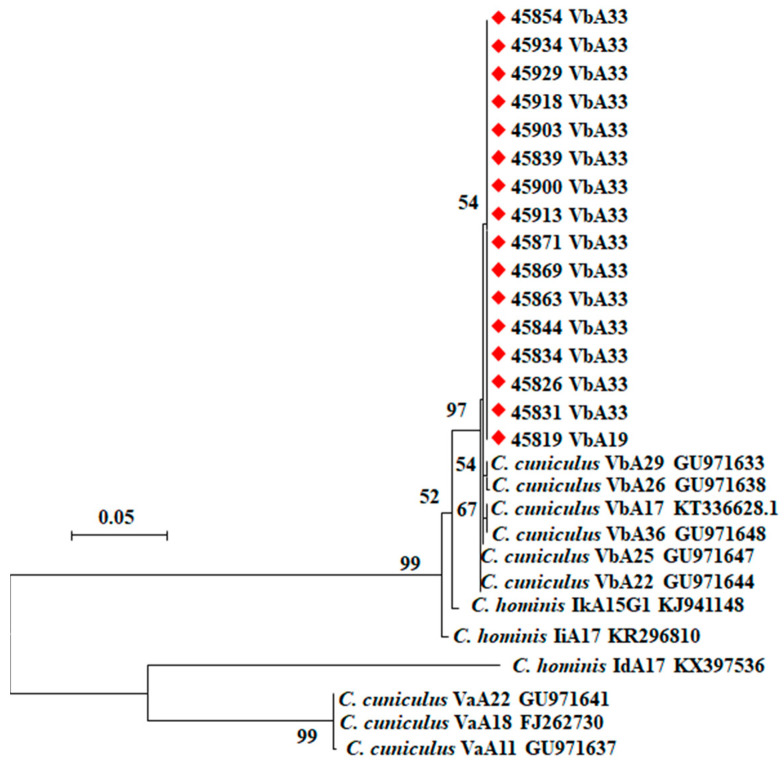
Phylogenetic tree of *Cryptosporidium* spp. based on *gp60* sequences through a maximum likelihood analysis based on substitution rates calculated with the general time reversible model. Numbers at the internodes represent bootstrap values (>70%) from 1000 replicates. The *Cryptosporidium cuniculus* samples identified in this study are labeled with red rhombus.

All 28 samples amplified by PCR analysis of the *SSU* rRNA gene had *C. cuniculus* by RFLP analysis (Figure 1). They produced two types of nucleotide sequences. Among them, ten sequences were identical to those in GenBank (AY120901, FJ262724, etc.), while two sequences had an A to T substitution near the 5′ end of the partial gene. In the phylogenetic analysis of the *SSU* rRNA sequences, *C. cuniculus* sequences obtained from the 12 samples clustered with reference sequences from GenBank (Figure 2). Of the 28 *C. cuniculus* samples, 16 were successfully subtyped by sequence analysis of the *gp60* gene, with two subtypes being identified: VbA19 (*n* = 1) and VbA33 (*n* = 15). These sequences were identical to each other in the non-repeat regions but had one A to T substitution compared to sequences (KU852732, GU097641, GU097647, GU971639, etc.) in GenBank (Figure 3). The VbA19 subtype was found only in a 6-month-old rabbit from farm El-Gharbia 3, while the VbA33 subtype was found on other *Cryptosporidium*-positive farms. Among the samples from four cages of animals with diarrhea, one sample from a cage of 4-month-old rabbits on El-Dakahlia 1 was positive for *C. cuniculus* VbA33 (Table 3).

## 3. Discussion

The results of the present study suggest a common occurrence of *Cryptosporidium* spp. in rabbits in the study areas. In this study, the overall occurrence of *Cryptosporidium* spp. in rabbits was 11.9% (28/235). This is similar to the infection rates of 11.2% (24/215) in a study of two rabbit farms in Heilongjiang Province, China [35], and 13.2% (14/106) in rabbits residing in Sydney drinking water catchments [36]. It is, however, higher than infection rates found in rabbits from Australia (6.8% or 12/176 and 8.4% or 22/263) [37,38], and Nigeria (3.7% or 4/107) [19], but lower than the infection rate recorded in pet rabbits in Japan (21.9% or 21/96) [16]. Several reports from China showed low infection rates of 1.03% (3/290), 2.4% (9/378), 3.4% (37/1081), and 3.4% (11/321) [13,39,40,41]. The differences in the infection rates of *Cryptosporidium* spp. among studies may be attributed to differences in sample size, rabbit breeds, management systems, geographic regions, and sample collection seasons. In one study, the infection rate of *Cryptosporidium* spp. in dead juvenile rabbits suffering from diarrhea was significantly higher than healthy ones (30.3% vs 3.3%) [16]. Among the nine farms examined in the present study, the occurrence of *Cryptosporidium* spp. on El-Gharbia 2 (24%) was higher than other farms (0–21%), possibly because of the poor hygiene and management practices on the farm.

Like in other animals, the infection rate of *Cryptosporidium* spp. is significantly higher in rabbits of youngest age. In this study, rabbits of <3 months had a significantly higher *Cryptosporidium* infection rate than older rabbits. Our findings are in agreement with observations in earlier studies, where the highest prevalence of *Cryptosporidium* spp. was recorded in young rabbits [13,39]. Similar age-associated occurrence of *Cryptosporidium* spp. has been reported in humans, cattle, and bamboo rats [33,34,42]. In the present study, a higher detection rate of *Cryptosporidium* spp. was recorded in Hi-Plus rabbits than Rex and New Zealand ones, possibly because of the high number of samples and sampling of many young animals. In contrast to our results, Rex and New Zealand rabbits were more susceptible than other breeds to *Cryptosporidium* infection in some earlier studies in China [13,35]. 

Generally, few clinical signs have been associated with cryptosporidiosis in rabbits, especially adult ones, and the infection is often not recognized due to the asymptomatic oocyst shedding [39,41]. This is in line with our results, where most rabbits were apparently healthy. Although two reports observed fatality in outbreaks of diarrhea in rabbits due to cryptosporidiosis [16,43], *Cryptosporidium* was detected in only one of the four samples from animals with diarrhea. 

All isolates of *Cryptosporidium* spp. detected in our study were genotyped as *C. cuniculus*, which is one of the causes of human cryptosporidiosis and has zoonotic significance [44]. In some countries, such as the UK, Australia and New Zealand, many sporadic cases of human cryptosporidiosis have been attributed to infections with *C. cuniculus* [22,24,25,45]. It was recognized as the third most important *Cryptosporidium* species causing cryptosporidiosis in humans in the UK during 2007 to 2008 and New Zealand during 2009 to 2019 [24,25]. It was also associated with a waterborne outbreak of cryptosporidiosis due to contamination of treated drinking water by wild rabbits [46,47]. Humans may be infected with *C. cuniculus* via contaminated water or direct contact with rabbits [22].

In this study, based on *gp60* sequence analysis, the *C. cuniculus* isolates belong to two subtypes (VbA19 and VbA33) in the Vb subtype family. Previously, the VbA19 subtype was isolated from rabbits in the Czech Republic [46], while the VbA33 subtype was detected in humans in the UK [48]. The two subtypes identified in the present study, however, differed from them by one nucleotide in the non-repeat region. Currently, Va and Vb are the only two subtype families within *C. cuniculus*. Between them, subtypes in the Va subtype family are more commonly seen in humans while those in the Vb subtype family are more commonly seen in rabbits [35]. The occurrence of similar subtypes of *C. cuniculus* in humans and rabbits supports the zoonotic potential of *C. cuniculus* [41].

In conclusion, to the best of our knowledge, this is the first study on the genetic identity of *Cryptosporidium* spp. in rabbits in Egypt. The results of this study suggest a common occurrence of *C. cuniculus* in farm rabbits in several areas of the country. The detection of *C. cuniculus* in this study supports the potential role of rabbits as a source of human infections. Further studies from other localities in Egypt are needed to improve our understanding of the clinical and public health significance of *Cryptosporidium* spp., in rabbits in Egypt.

## 4. Materials and Methods

### 4.1. Ethics Statement

Permission was obtained from the owners of the farms before collection of fecal specimens. All fieldwork associated with this study was conducted in compliance with the Guide for the Care and Use of Laboratory Animals in Egypt. The study protocol was approved by the Ethics Committee of the Faculty of Veterinary Medicine, Mansoura University, Egypt.

### 4.2. Specimen Collections

A total of 235 fresh fecal specimens were collected between July 2015 and April 2016 from nine rabbit farms randomly selected in El-Dakahlia, El-Gharbia, and Damietta provinces in Egypt. All farms sampled in this study were medium-sized farms housing 700–900 rabbits. Fecal specimens were randomly collected from at least 20% of rabbit cages on each farm. Each specimen consisted of 3–5 fresh fecal pellets gathered from each cage. Each collection from each cage (containing 4–7 rabbits) was regarded as one specimen. The fecal pellets were placed into a sterile disposable plastic bag labeled with the age and breed of the animals and sampling date and location. Animals in four cages showed clinical signs of enteric diseases (emaciation, dehydration, and diarrhea) at the time of specimen collection. The rabbits were divided into three age groups: <3-month-old, 3–6-month-old, and >6-month-old. Specimens were stored in 70% ethanol at 4 °C until being transported to the Centers for Disease Control and Prevention, Atlanta, Georgia, USA for DNA extraction and molecular analysis. 

### 4.3. DNA Extraction and PCR Amplification

The fecal specimens were washed twice with distilled water by centrifugation to remove ethanol before DNA extraction. Extraction of genomic DNA from specimens was performed using the FastDNA SPIN Kit for Soil (BIO 101, Carlsbad, CA, USA). The genomic DNA was eluted with 100 μL reagent-grade water and stored at −20 °C until PCR analysis.

### 4.4. Cryptosporidium Detection, Genotyping and Subtyping

*Cryptosporidium* spp. in the specimens were detected by nested PCR analysis of a ∼830-bp fragment of the small subunit rRNA (*SSU rRNA*) gene as previously described [49]. *Cryptosporidium* species were identified by restriction fragment length polymorphism (RFLP) analysis of the secondary PCR products of *SSU rRNA* gene using *Ssp*I (New England BioLabs, Ipswich, MA, USA) and *Vsp*I (Promega, Madison, WI, USA) restriction enzymes [50]. All *Cryptosporidium*-positive specimens were selected for further subtyping by PCR and sequence analysis of the 60-kDa glycoprotein (*gp60*) gene [51]. Each specimen was analyzed twice for each genetic target, using *C. baileyi* DNA as the positive control for the *SSU rRNA*-based PCR, *C. parvum* DNA as the positive control for *gp60*-based PCR, and reagent-grade water as the negative control for both PCR assays. 

### 4.5. DNA Sequence and Phylogenetic Analysis

Montage PCR filters (Millipore, Bedford, MA, USA) were used to purify all secondary PCR products of both genes. The purified products were sequenced in both directions using the secondary PCR primers and Big Dye^®^ Terminator v3.1 Cycle Sequencing Kit (Applied Biosystems, Foster City, CA, USA) on an ABI 3130 Genetic Analyzer (Applied Biosystems). ChromasPro (version 1.5) (www.technelysium.com.au/ChromasPro.html/, accessed on 22 June 2009) was used to edit and assemble the DNA sequences, while ClustalX 2.0.11 (http://www.clustal.org/, accessed on 1 June 2018) was used to align the obtained nucleotide sequences against each other and reference sequences from GenBank to determine the genetic relatedness of various *C. cuniculus* subtype families. A phylogenetic tree was constructed using the maximum likelihood algorithm implemented in MEGA version 7.0.26 (www.megasoftware.net/, accessed on 1 May 2017) based on substitution rates calculated with the general time reversible model. Bootstrap analysis was applied to evaluate the reliability of cluster formation in the phylogenetic tree with 1000 replicates.

### 4.6. Statistical Analysis

Differences in infection rates of *Cryptosporidium* spp. among rabbits of different age groups, localities, and breeds were estimated using the Fisher’s exact test. The SPSS software version 20.0 (IBM, Armonk, NY, USA) was used in the statistical analysis of the data. Differences were considered significant at *p* ≤ 0.05.

## Figures and Tables

**Table 1:** Distribution of *Cryptosporidium* spp. in rabbits by farm and age group in El Dakahlia, El-Gharbia, and Damietta provinces, Egypt.

Farm	Age (Month)	No. of Samples	*Cryptosporidium* spp. *		
			No. Positive (%)	95% Confidence Interval	
				Lower Limit	Upper Limit
El-Dakahlia 1	<3	10	2	-	-
	3–6	11	1	-	-
	>6	10	1	-	-
	Subtotal	31	4 (13%)	1.10	24.69
El-Dakahlia 2	<3	9	2	-	-
	3–6	7	2	-	-
	>6	7	0	-	-
	Subtotal	23	4 (17%)	1.90	32.89
El-Dakahlia 3	<3	11	3	-	-
	3–6	11	2	-	-
	>6	6	1	-	-
	Subtotal	28	6 (21%)	6.20	36.59
El-Gharbia 1	<3	8	0	-	-
	3–6	8	0	-	-
	>6	10	0	-	-
	Subtotal	26	0 (0%)	0.00	0.00
El-Gharbia 2	<3	7	4	-	-
	3–6	12	2	-	-
	>6	6	0	-	-
	Subtotal	25	6 (24%)	7.25	40.74
El-Gharbia 3	<3	12	2	-	-
	3–6	11	1	-	-
	>6	7	0	-	-
	Subtotal	30	3 (10%)	−0.73	20.73
Damietta 1	<3	9	1	-	-
	3–6	10	0	-	-
	>6	8	0	-	-
	Subtotal	27	1 (4%)	−3.42	10.82
Damietta 2	<3	4	1	-	-
	3–6	8	1	-	-
	>6	8	0	-	-
	Subtotal	20	2 (10%)	−3.14	23.14
Damietta 3	<3	9	1	-	-
	3–6	8	1	-	-
	>6	8	0	-	-
	Subtotal	25	2 (8%)	−2.63	18.63
Total	-	235	28 (11.9%)	-	-

* Fisher’s exact test: *t* = 12.258, *p* = 0.106.

**Table 2:** Factors associated with *Cryptosporidium* infection in rabbits in Egypt.

Factors		Sample Size	*Cryptosporidium* spp.				
			No. Positive (%)	95% Confidence Interval		Fisher’s Exact Test	*p*
				Lower Limit	Upper Limit		
Age (month)	<3	79	16 (20)	11.38	29.11	11.237	0.003
	3–6	86	10 (12)	4.85	18.40		
	>6	70	2 (3)	−1.03	6.79		
Breed	Rex	57	4 (7)	0.30	13.60	2.283	0.333
	Hi-Plus	98	15 (15)	8.10	22.40		
	New Zealand	80	9 (11)	4.30	18.20		
Locality	El-Dakahlia	82	14 (17)	8.90	25.20	3.685	0.157
	El-Gharbia	81	9 (11)	4.20	17.90		
	Damietta	72	5 (7)	1.00	12.70		

**Table 3:** Distribution of 16 *Cryptosporidium cuniculus* subtypes at the *gp60* locus in rabbits in Egypt.

Sample ID	*C. cuniculus* Subtype	Age (Month)	Location	Breed
45819	VbA19	6	El-Gharbia 3	Hi-Plus
45826	VbA33	1	El-Dakahlia 1	Rex
45831 *	VbA33	4	El-Dakahlia 1	Rex
45834	VbA33	4	Damietta 3	New Zealand
45839	VbA33	3	Damietta 2	Hi-Plus
45844	VbA33	4	El-Gharbia 2	Hi-Plus
45854	VbA33	3	El-Gharbia 2	Hi-Plus
45863	VbA33	2	El-Gharbia 2	Hi-Plus
45869	VbA33	2	El-Gharbia 2	Hi-Plus
45871	VbA33	1	El-Gharbia 2	Hi-Plus
45900	VbA33	1	Damietta 3	New Zealand
45903	VbA33	2	El-Dakahlia 3	New Zealand
45913	VbA33	1	El-Dakahlia 3	New Zealand
45918	VbA33	1	El-Dakahlia 3	New Zealand
45929	VbA33	1	El-Dakahlia 2	Hi-Plus
45934	VbA33	2	El-Dakahlia 2	Hi-Plus

* From rabbit with diarrhea.

## Data Availability

All relevant data are within the paper.
